# CCD2MD: A Suite of Packages for Preparing Co-Folded Outputs for Molecular Dynamics Simulations

**DOI:** 10.1021/acs.jcim.5c02066

**Published:** 2025-11-05

**Authors:** Katarina E. Blow, Matyas Parrag, Phillip J. Stansfeld

**Affiliations:** † School of Life Sciences, Gibbet Hill Campus, 2707 University of Warwick, Gibbet Hill Road, Coventry CV4 7AL, U.K.; ‡ Department of Chemistry, 2707 University of Warwick, Gibbet Hill Road, Coventry CV4 7AL, U.K.

## Abstract

Protein–lipid interactions play a crucial role in the stability and function of membrane proteins. While experimental approaches to characterize these interactions in a native-like membrane environment can be challenging, computational techniques offer a powerful alternative for identifying and analyzing potential binding sites. Recent advances in cofolding methods now enable the prediction of holo protein structures, capturing conformational changes that may occur upon lipid binding and thereby improving the accuracy of binding site characterization. However, the outputs from these methods often require postprocessing to ensure compatibility with widely used molecular dynamics force fields. In this work, we introduce CCD2MD, a modular toolkit designed to convert cofolding outputs into simulation-ready systems for GROMACS. CCD2MD supports both atomistic and coarse-grained representations with or without membrane embedding. While CCD2MD is exemplified here with protein–lipid systems, its modular design allows for straightforward adaptation to other cofolded biomolecular assemblies, incorporating complexes with nucleic acids, small molecules, carbohydrates, or metal ions, thereby enabling a variety of simulation setups across multiple scales.

## Graphical abstract

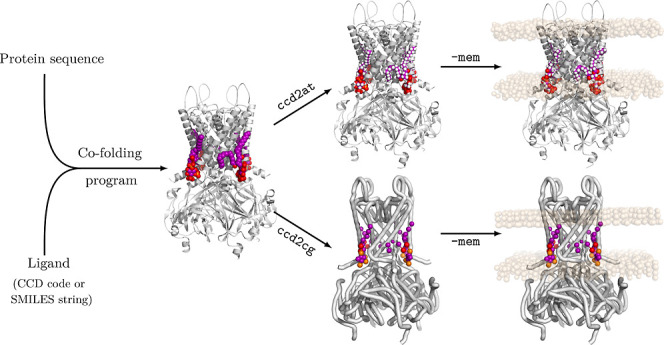

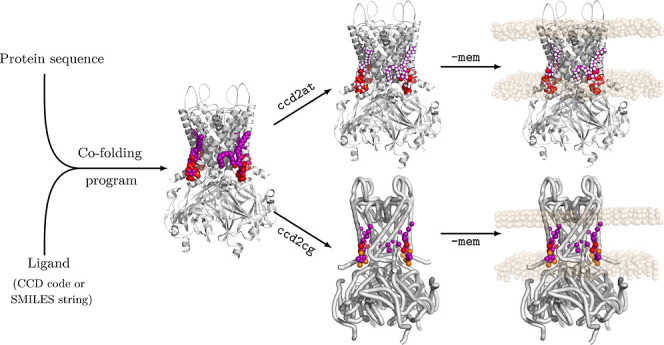

## Introduction

Interactions between proteins and small-molecule ligands are central to many biological processes. In particular, lipid binding can modulate and regulate the membrane protein function,^1,2^ while the efficacy of certain antimicrobials arises from their ability to disrupt protein–ligand interactions.^3–5^ Computational approaches offer valuable tools to investigate and characterize these interactions, especially when experimental data are limited. In silico methods can assess the stability of binding modes and provide dynamic insights, enabling the virtual screening of potential binders.

Molecular dynamics (MD) simulations are commonly used for this purpose, offering detailed views of protein flexibility and movement. These simulations can be performed using either fully atomistic (all-atom, AA) or coarse-grained (CG) representations.^5,6^ However, the reliability of such simulations depends heavily on the quality of the starting structure,^6^ as proteins may undergo conformational changes upon ligand binding.^7^ Docking ligands to apo states—or to incorrect holo conformations—can result in poor binding poses,^8^ leading to molecular models and subsequent simulations that fail to capture the true dynamics of the complex.

Co-folding programs, such as RosettaFold All-Atom,^9^ AlphaFold3,^10^ Chai-1,^11^ Boltz-1,^12^ and Protenix,^13^ enable the generation of ligand-bound protein structures, with ligands represented either via CCD codes or SMILES strings. These approaches have shown promising accuracy in reproducing known protein–ligand complexes,^1,9–13^ marking a significant advancement in the prediction of arbitrary bound states. However, chirality issues remain widespread across both software packages^10,13^ and molecular representations, likely due to biases in training data favoring ligands of opposite chirality.^14,15^ While SMILES strings offer flexibility for representing any small molecule, CCD codes, though more limited in scope, tend to better preserve molecular chirality.^15^ They also provide consistent and unique representations across platforms. The integration of user-defined CCD codes into the AlphaFold3 (AF3) distribution extends these benefits to arbitrary molecules, enhancing the accuracy and interoperability of cofolded protein–ligand models.

Despite known chirality issues, cofolding approaches remain powerful tools for investigating protein–ligand interactions (such as a recent work indicating their use in the interpretation of cryo-EM maps,^16^ and their applicability to guide investigations into protein-sugar interactions^17^). However, significant postprocessing is often required before these structures can be used as starting points for molecular simulations. One common challenge is the mismatch between ligand representations: CCD codes used in cofolding outputs may not align with naming conventions or atom ordering in force fields, such as CHARMM. This issue is further complicated by inconsistencies across force fields and cofolding software, particularly when SMILES strings are used. For SMILES-based inputs, atom naming and ordering typically follow the sequence of atoms in the input string. Because SMILES representations are not unique, different strings describing the same molecule can result in different atom orders, which may not match the force field definitions. As a result, converting cofolding outputs into simulation-ready systems often requires either generating new force field files for the cofolded output’s atom naming and numbering using tools like CHARMM-GUI,^18^ or manually reorganizing and relabeling atoms; both of which can be time-consuming and error-prone.

To facilitate the use of cofolding outputs in molecular dynamics (MD) simulations within GROMACS,^19^ we introduce the CCD2MD distribution (github.com/keb721/CCD2MD). This toolkit includes a suite of packages: pos2cif, ccd2at, and cd2cg, and for completeness, at2ccd, at2cg, and at2mem, designed to automate the conversion of cofolded protein–ligand structures into formats compatible with the CHARMM36 ^20^ (atomistic, referred to hereafter as CHARMM) and Martini 3 ^21^ (coarse-grained, referred to as Martini) force fields. The distribution also includes a membrane insertion pipeline based on MemPrO,^22^ enabling the embedding of proteins with post-translational modifications or bound ligands into lipid bilayers for further simulations. A schematic overview of this workflow is shown in Figure 1.

**Figure 1:**
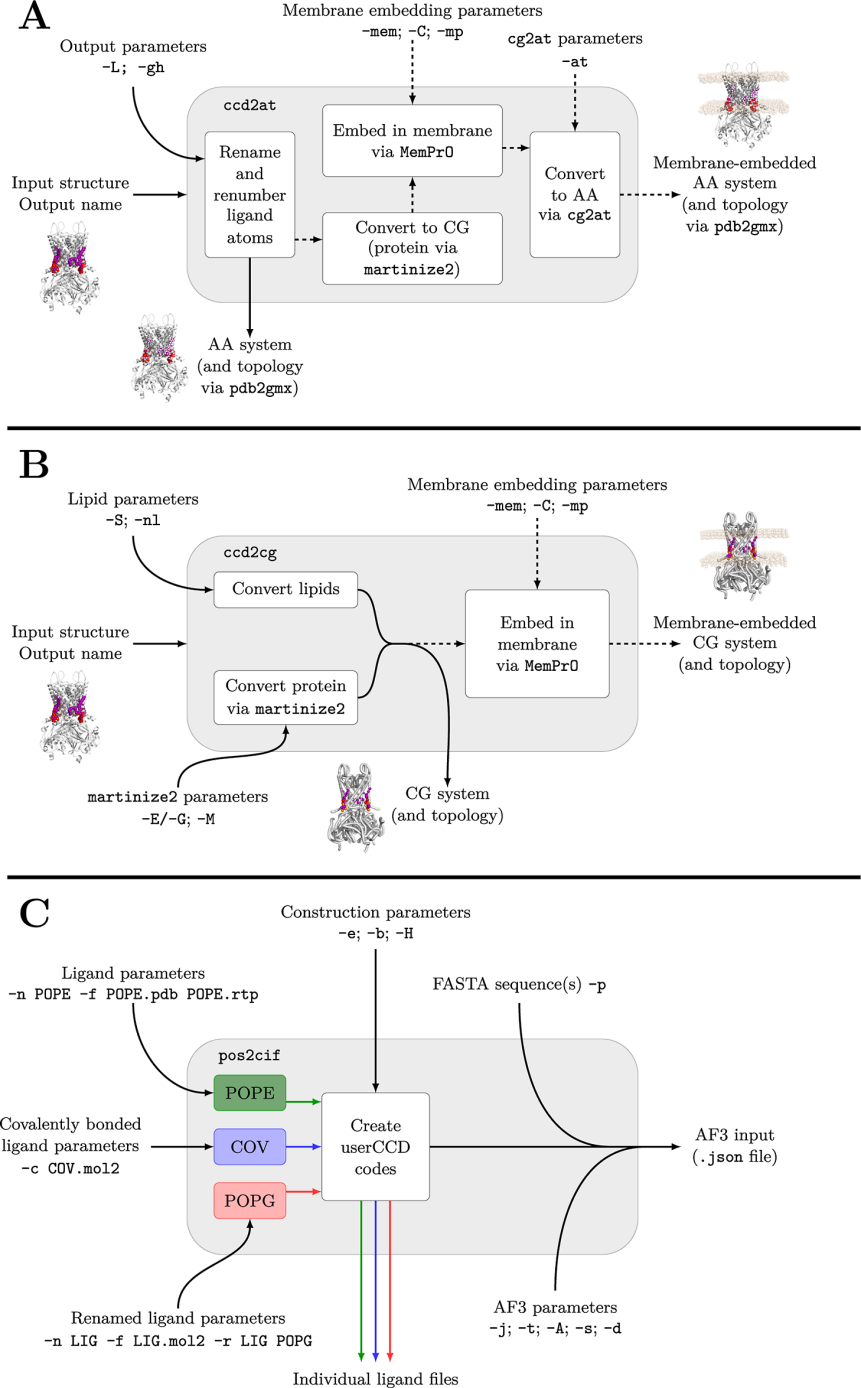
Example workflows enabled by CCD2MD via ccd2at (**A**), ccd2cg (**B**), and pos2cif (**C**). In ccd2at and ccd2cg, the input structure from cofolding (here Kir 2.2, UniProt ID: Q14500, with POP2, see Table 1) and various additional parameters are used to give outputs of systems and topologies. Optionally, the converted structure may be embedded in a membrane (dashed lines, requiring the -mem flag). In pos2cif, various ligand inputs (including covalently bonded ligands) are input to get the userCCD codes for the input ligands and an output json file compatible with AlphaFold3.

For molecules in CCD2MD which are also in the CCD database, either ccd2at or ccd2cg can be used to convert the output of any cofolding code, which allows the input of CCD codes (e.g., Chai-1 ^11^ and Protenix^13^) into CHARMM or Martini formats. Alternatively, it is possible for an end-user to set up these conversions from defined SMILES codes. The procedure for this is detailed in the Supporting Information. User-defined CCD codes (mapped to CHARMM outputs) for every molecule in CCD2MD are presented in CCD2MD.cif, with pos2cif allowing users to create their own user-defined CCD files.

Below we will outline the methodology and use of the code base, with the aim that this will allow for ease of user-extensibility to additional ligands. This includes an overview of atomistic conversions through ccd2at and at2ccd. We then discuss the generation of user-defined CCD codes via pos2cif and the capability to produce an entire AF3 input file. An overview of coarse-grained conversion via ccd2cg and at2cg is then presented. The membrane insertion pipeline (via MemPrO^22^) is discussed, and code dependencies are given. Finally, we give a broad overview of the code and plans for future development.

## Methodology

Table 1 gives the list of ligands present in CCD2MD along with their CHARMM and Martini names and their CCD codes where applicable. CCD codes have been presented (in addition to user-defined CCD information in CCD2MD.cif) to allow for the use of open-source cofolding packages. Conversion information for the ligands present in CCD2MD (excluding hydrogen atoms) is provided in the Ref_data folder within the distribution, with user-defined CCD codes in the CCD2MD.cif file. We note that this table includes several ligands, which are non-native in CHARMM, and several ligands where the CCD2MD version differs from the default CHARMM version. CCD2MD, therefore, includes charmm36-ccd2md.ff, which includes all necessary.rtp and .hdb files for utilizing these molecules within CHARMM. While this database has a focus on lipids, the modular design of CCD2MD is intended to allow for the user to easily extend this framework to other ligands of interest.

**Table 1:** Ligands Present in CCD2MD^q^

CHARMM	CCD	Martini
lipids		
CARD^a^ ^,^ ^n^	–	CARD^p^
CHL1	CLR	CHL1^p^ ^,^ ^23^
DGPC	–	DGPC^p^
DGPE	–	DGPE^p^
DLPC	–	DLPC^p^
DLPE	WNZ	DLPE^p^
DMPC	MC3	DMPC^p^
DMPE	46E	DMPE^p^
DMPI	–	DMPI^p^
DMPS	–	DMPS^p^
DNPC	–	DNPC^p^
DNPE	–	DNPE^p^
DOPC	–	DOPC^p^
DOPE	–	DOPE^p^
DOPS	17F	DOPS^p^
DPG3^m^	–	DPG3
DPPC	PCF	DPPC^p^
DPPE	–	DPPE^p^
DYPC	–	DYPC^p^
DYPE	–	DYPE^p^
LIP1^m^	–	LIP1
LIP2^m^	–	LIP2
LIP3^m^	–	LIP3
LIPA	–	LIPA
POP1^b^	–	POP1^p^ ^,^ ^24^
POP2^c^ ^,^ ^n^	–	POP2^p^ ^,^ ^24^
POP3^d^ ^,^ ^n^	–	POP3^p^ ^,^ ^24^
POP4^e^	–	POP4^p^ ^,^ ^24^
POP5^f^	–	POP5^p^ ^,^ ^24^
POPI6^g^ ^,^ ^n^	–	POP6^p^ ^,^ ^24^
POP7^h^ ^,^ ^n^	–	POP7^p^ ^,^ ^24^
CHARMM	CCD	martini
POPA	D21	POPA^p^
POPC	POV	POPC^p^
POPE	6OU	POPE^p^
POPG	PGW	POPG^p^
POPI	–	POPI^p^ ^,^ ^24^
POPS	D39	POPS^p^
RAMP^m^	–	RAMP
REMP^m^	KDL	REMP
SSM1^i^	–	SSM1^p^
TMM1^m^	–	TMM1
TMMA^m^	–	TMMA
UDP1^m^	5TR	UDP1
UDP2^j^	–	UDP2
post-translational modifications		
CYSD	–	CYSD^25^
CYSF	–	CYSF^26^
CYSG	–	CYSG^26^
CYSP	–	CYSP^26^
CYST	–	CYST^25^
GLYM	–	GLYM^26^
detergents		
BDDM^o^	LMT	–
BLMN^k^ ^,^ ^o^	LMN	–
BOG1^l^ ^,^ ^o^	BOG	–

a POCL2 in CHARMM36.

b POPI13 in CHARMM36.

c POPI2D in CHARMM36.

d POPI34 in CHARMM36.

e POPI14 in CHARMM36.

f POPI15 in CHARMM36.

g POPI24 in CHARMM36.

h POPI2A in CHARMM36.

i SSM in CHARMM36.

j UNDPP in CHARMM36.

k BLMNG in CHARMM36.

l BOG in CHARMM36.

m Non-native in CHARMM36.

n Alternative choice of headgroup charge.

o Renamed atoms.

p New lipidome mapping available^27^.

q The CCD column gives the CCD code, where applicable. The CHARMM column gives the CHARMM name, where indicated there have been extensions to the default CHARMM distribution (^*m*^) or minor changes to CHARMM names, shown in footnotes. The Martini column gives the Martini name.

The CCD2MD atomistic converters, ccd2at and at2ccd, allow for the automated renaming and reordering of ligand atoms and residues between those from the CCD code and the CHARMM distribution. They read as the input either a .pdb, .gro, or.cif file and output the new ligands in an ordered .pdb file.

Additional user-defined CCD codes reflecting the representation of ligands in a force field of interest can be created using pos2cif, which creates .cif files and a .json file containing a userCCD field for input into AF3 for an arbitrary number of specified input ligands, where positions are provided in .crd, .gro, .mol2, or.pdb files. Optionally, .itp, .rtf, or.rtp files may be added for a more accurate determination of charges and bonding information. Optionally, protein information can be added to create a .json file, which can be used directly with AF3. Where there is an overlap between the specified name and existing CCD codes, the user-defined CCD will supersede this. The ability to specify atom orderings and names means that, unlike the rest of the CCD2MD suite, pos2cif should be easily useable with different all-atom force fields by specifying files relevant to the force field of interest. Both ligands and amino acid modifications can be converted in this way.

The CCD2MD CG converters, ccd2cg and at2cg, read as input files either a .cif, .gro, or.pdb file, which can contain multiple CCD ligands. The output is a reordered .pdb file. It also optionally takes in extra flags to be passed to Martinize2,^28^ while creating the CG representation of the protein. The atomic mappings used for the ligands are based on CG2AT^29^ and include by default the latest reparameterizations of cholesterol,^23^ phosphatidylinositols,^24^ and lipidation post-translational modifications.^26^ Mappings can be found in the CG2AT-CCD2MD folder on GitHub (github.com/keb721/CCD2MD).

### Atomistic Conversion

Atomistic conversion using ccd2at and at2ccd (e.g., for cholesterol) is performed by comparing atom naming and ordering between the CCD output (e.g., CLR_CCD.txt for CCD code CLR) and the CHARMM force field representation (e.g., CHL1_CHARMM.txt). Where discrepancies exist, a mapping file (e.g., CHL1.txt) defines the correspondence between atom names. A master file, database.csv, provides a reference linking CCD codes to their CHARMM and Martini equivalents.

By default, ligands are not assigned to a separate chain, and residue numbering continues with any preceding protein. This behavior can be modified using the -L or --ligchain command-line option. Chains are processed alphabetically, with empty chains handled last. Because cofolding techniques typically omit hydrogen atoms, any hydrogens present in input files (e.g., .cif files from the CCD database) are stripped. Hydrogens are reintroduced during topology generation using either pdb2gmx in GROMACS,^19^ or CG2AT-CCD2MD^29^ if membrane embedding is applied.

#### Usage of ccd2at

ccd2at INPUT OUTPUT [-L] [-S < smiles>···] [-gh < pdb2gmx >]

- INPUT: a .cif, .gro, or.pdb file containing one or more CCD ligands (including user-defined entries from CCD2MD.cif).
- OUTPUT: the name of the resulting .pdb file with CHARMM-compatible atom naming and ordering.
- Ligands not found in the CCD2MD database remain unchanged.
- The -L flag assigns ligands to a separate chain.
- The -S or --SMILES flag allows conversion from user-defined SMILES strings (see the Supporting Information).
- The -gh/--pdb2gmx flag passes additional arguments to pdb2gmx. This overrides all defaults.

If membrane embedding is used, the system topology (topol.top) and hydrogen placement are handled by CG2AT-CCD2MD. Otherwise, pdb2gmx is used to generate the topology and add hydrogens, producing OUTPUT_H.pdb.

#### Usage of at2ccd

at2ccd INPUT OUTPUT [-L]

- INPUT: a .cif, .gro, or.pdb file.
- OUTPUT: a .pdb file with CHARMM ligands renamed and reordered according to CCD conventions.
- By default, ligands are not assigned to a chain and residue numbering continues from the previous protein.
- The -L flag assigns ligands to a separate chain.

### User CCD Codes

With the release of AlphaFold 3, the option to create arbitrary user-defined CCD (userCCD) codes removes the requirement for using either a SMILES string or known CCD code to add a ligand to a cofolding prediction. CCD2MD includes CCD2MD.cif (which contains userCCD codes (matched to the CHARMM naming and ordering) for all molecules in the database) as well as pos2cif, which creates .cif files (with userCCD mappings) and a .json file for input into AF3 for an arbitrary number of specified input ligands if provided with ligand name(s) and position file(s). Optionally, file(s) containing bonding information may be added for a more accurate determination of charges and covalent interactions. Where CHARMM/Martini names overlap with existing CCD codes, provided userCCDs will supersede these. We note that the ability to specify atom orderings and names means that unlike the rest of the CCD2MD suite, pos2cif should be useable with different all-atom force fields by specifying files relevant to the force field of interest. Both ligands and amino acid modifications can be converted in this way.

pos2cif takes in optimal ligand coordinates and produces single-chain single-residue positions for these. It may also take in a ligand made of the covalent bonding of several smaller ligands and can be used to create userCCD files for a combination of these. Amino acid modifications (see post-translational modifications in Table 1) can be added by defining a single-chain single-residue ligand of the modified amino acid and adding the chosen userCCD code as a post-translational modification to the relevant residue; this can be used to define noncanonical amino acids. Examples of post-translational modifications are presented in Figure 2.

**Figure 2:**
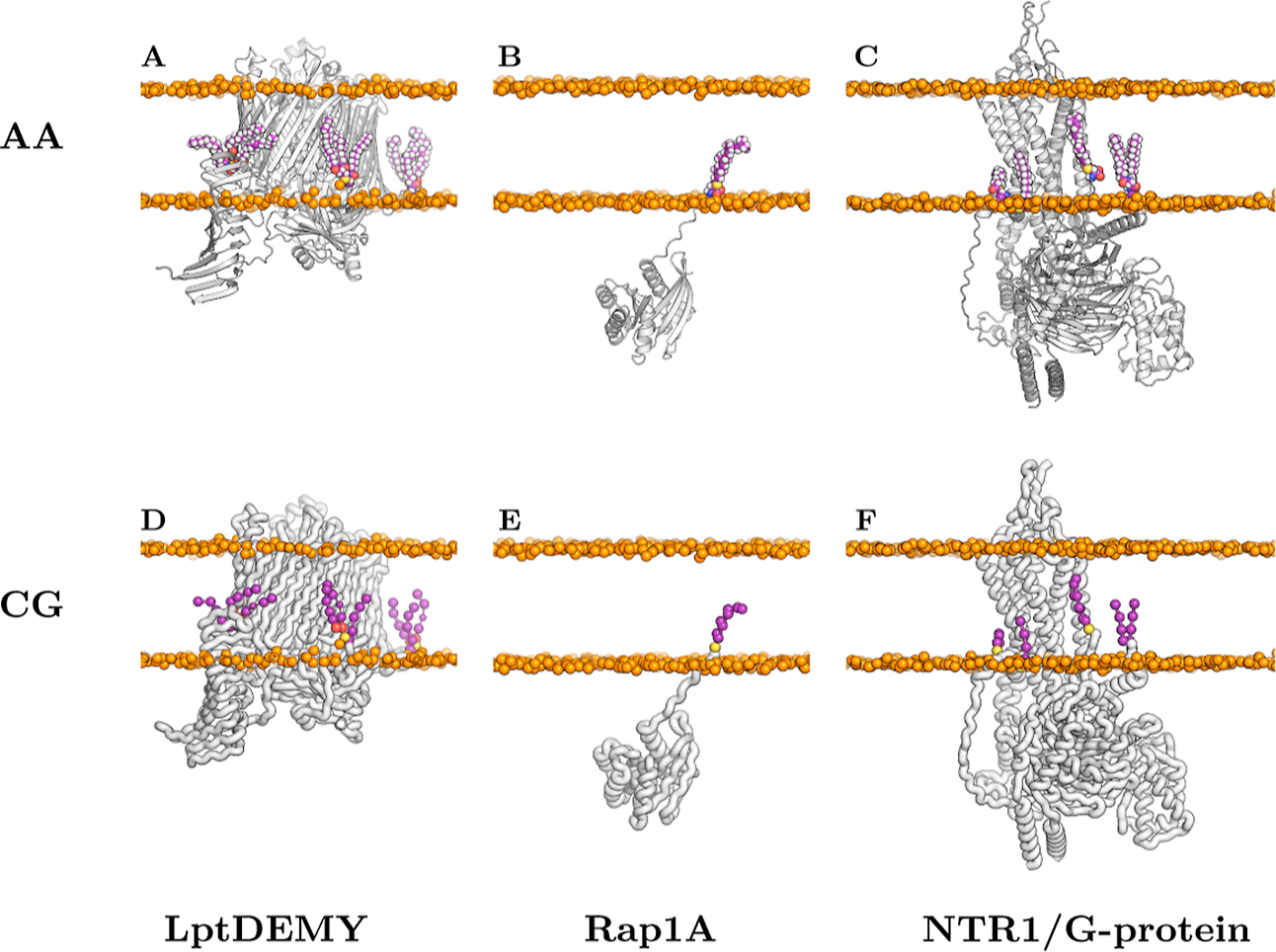
Modified protein complexes from AlphaFold3 ^10^ utilizing user-defined CCD codes (created via CCD2MD). For each complex, both atomistic (CHARMM, top row) and coarse-grained (Martini, bottom row) representations are shown. (**A** (atomistic) and **D**) (coarse-grained) LptDEMY (Uniprot IDs P31554, P0ADC1, P0ADN6, and P31063) with N-terminal triacylcysteine modifications. (**B** and **E**) Rap1A (P62834) with a C-terminal cysteine geranylgeranyl modification. (**C** and **F**) Neurotensin receptor (NTR1) G-protein complex (P30989, P63096, P62873, and P59768) featuring both cysteine geranylgeranyl and cysteine palmitoyl modifications.

For converting single-chain single-residue ligands, pos2cif reads as input files either.crd, .gro, .mol2, or .pdb file(s) to determine the ligand position. For .crd, .gro, or.pdb inputs, there may be multiple different components, but there must be only one copy of the ligand of interest (this should be its canonical configuration). Bonding information and information on atomic charges can optionally be provided through .itp, .rtf, or .rtp files (bonding information is already present in .mol2 files). If not provided, bonding information is inferred by atom proximity, using a hard cutoff to determine if two atoms are close enough to be bonded. The type and aromaticity of the bond are required by AF3 but are not used to generate the model, so are set to single nonaromatic bonds without a loss of function. Single-chain single-residue ligands can be renamed by adding a flag of the form -r/–rename < old> < new>, where old is the name of the ligand in the position and bonding files and new is the desired output name.

For converting covalently bonded ligands, pos2cif reads as input files either a .mol2 file, which contains both the position and bonding information, or a pair of .pdb and .itp files; unlike above, each pair of files for a single molecule must immediately follow in the .pdb then .itp order. For all file types, only the covalently bonded ligand can be present. Where a covalently bonded ligand has multiple copies of the same component, the coordinates of the first instance of the component are taken as the ideal coordinates. Charge and bond types are assigned as described above. The names of these ligands cannot be changed via the -r/–rename flag.

The use case of pos2cif is as follows:

pos2cif [-n <ligand>··· -f <file>···]

[-r (<old> < new>) ...]

[-c ((<pdb> < itp>) | < mol2>) ...]

[-e < charge>] [-b < bond>] [-H]

[-j < json>] [- *t* < title>] [-A < afvers>]

[-s < seed>···] [-d < dialect>]

[-p (<protein> | (<protein> < *N* > )) ...]

-n/--names: specifies the codes for the ligands to be transformed, as given in the input files.
-f/--files: lists the input files (.crd, .itp, .mol2, .pdb, .rtf, .rtp) containing positional and optionally bonding information for the ligands.
-r/--rename < old> < new>: renames ligands, where <old> is the code in the input files and <new> is the desired output name.
-c/--covalent: adds covalently bonded ligands via either.mol2 files or pairs of.pdb and.itp files.
-e/--charge: sets a cutoff for rounding partial charges to integer values in the CIF file (default: 0.75 *e*).
-b/--bond: defines a distance cutoff (default: 1.4 Å) for generating bonds from positional data, used only when bonding information is not provided.
-H/--Hydrogen: outputs hydrogen atoms and bonding; for default hydrogen stripping, consistency across input files is required.
-j/--json: specifies the name of the AF3 input.json file containing userCCD data (default: output.json). Individual ligand CIF files (NAME_output.cif) are also generated.
-d/--dialect: sets the dialect for the.json file (default: alphafold3).
-A/--afvers: specifies the AlphaFold version (default: 2).
-t/--title: sets the system name used by AF3 (default: pos2cif_system).
-s/--seeds: specifies model seeds (default: 1).
-p/--protein: adds protein sequences via FASTA sequences. For *N* copies, users may pass *N* FASTA sequences, a single FASTA followed by *N*, or a combination of FASTA sequences and numerals.

AF3 outputs containing userCCD codes can be used alongside conventional CCD codes. Depending on the system components, the resulting models can be postprocessed using any of ccd2at, at2mem, at2cg, or ccd2cg. Conversion via ccd2at/ccd2cg is enabled by defining a “user” mapping that links userCCD codes to CHARMM-compatible atom names. Care should be taken to avoid naming conflicts, particularly if a userCCD code matches an atom name in the default CHARMM distribution.

### Coarse-Grained Conversions

For conversion to a coarse-grained (CG) representation, CCD2MD includes mapping files, such as CHL1_CG.txt, which define Martini beads and their contributing atoms. These atoms are referenced using CHARMM names and, where applicable, CCD and SMILES atom names. Ligands can be converted directly from cofolding CCD outputs to CG format by placing beads at the center of mass of their constituent atoms, excluding hydrogens (which are not present in cofolding outputs). While the absence of hydrogens may slightly affect bead placement, this impact is expected to be negligible, particularly after energy minimization.

Protein conversion with post-translational modifications (PTMs) is handled via Martinize2,^28^ with user-defined arguments optionally passed to the tool. The system topology (topol.top) and all relevant.itp files are written to the output directory. This methodology could be extended to other CG force fields provided suitable mapping files are available.

Although Martinize2 supports nonprotein components, it typically requires custom library and mapping files. CCD2MD bypasses this by including predefined mappings. Ligands are output without chain information. If the input is a.cif file, Martinize2 version 0.14.0 can process it directly, provided that the PyCifRW [Fn fn1] package is installed. Otherwise, an intermediate file (OUTPUTNAME_convert.pdb) is created for protein conversion. The CG protein and its topology are written as OUTPUTNAME_proteinCG.pdb and OUTPUTNAME_proteinCG.top, respectively, with the full system in OUTPUTNAME.pdb. While intermediate files are placed in the output directory, the topology and.itp files from Martinize2 remain in the working directory.

#### Usage of ccd2cg

ccd2cg INPUT OUTPUT [-S < smiles>···] [-nl]

[(-E [<elastic>]) | (-G < go>)]

[-M < martinize>]

- INPUT: a.cif, .gro, or.pdb file containing one or more CCD ligands.
- OUTPUT: the name of the output.pdb file in Martini format.
- Ligands not found in the CCD2MD database remain unchanged.
-S/--SMILES: allows conversion of user-defined SMILES strings (see Supporting Information).
-nl/--newlipidome: uses updated Martini 3 lipidome mappings.^27^
-E/--elastic: applies an elastic network for secondary structure bias.
-G/--go: applies a Go̅ network instead of an elastic network.
-M/--martinize: passes additional arguments to Martinize2

#### Usage of at2cg

at2cg INPUT OUTPUT [-nl]

[(-E [<elastic>]) | (-G [<go>])]

[-M [<martinize>]]

- INPUT: a.cif, .gro, or.pdb file.
- OUTPUT: a.pdb file containing the CG representation of the protein and any CHARMM ligands in the database.
-nl/--newlipidome: uses updated Martini 3 lipidome mappings.^27^
-E/--elastic or -G/--go: applies secondary structure biasing via elastic or Go̅ networks.
-M/--martinize: passes additional arguments to Martinize2

By default, CCD2MD assumes the generation of a secondary structure biasing network. If neither -E nor -G is specified, then an elastic network is applied. Users should avoid passing secondary structure biasing information via -M, as this is outside the scope of the current implementation. Additionally, when PTMs are present, the internal default is to pass -resid input to Martinize2, unless -resid mol is explicitly provided by the user.

### Membrane Insertion

To accurately simulate transmembrane proteins in their native environment, it is necessary to embed them within a representative membrane. CCD2MD facilitates this through integrated use of MemPrO^22^ for membrane orientation and embedding. This pipeline can also be used for membrane-associated proteins. Membrane insertion or association is supported by ccd2at, ccd2cg, at2cg, and at2mem, although embedding is currently not supported for ligands lacking a CG representation or proteins linked via a Go̅ network, as these are incompatible with MemPrO.^22^

For atomistic systems, the CG output from MemPrO^22^ is converted back to atomistic using CG2AT-CCD2MD, a modified version of CG2AT^29^ included in the CCD2MD GitHub distribution. Due to the CG-to-AA conversion, slight differences in ligand positioning may occur between the raw cofolding output and the membrane-embedded structure.

#### Usage

Membrane embedding is triggered by adding the -mem/–membrane flag to the standard command-line arguments. For example:

ccd2at INPUT OUTPUT [-S < smiles>···]

[-mem [<membrane>] [-C < conc>] [-mp < mempro>]]

[-at < cg2at>]

-mem/--membrane: specifies membrane composition using the format from Insane4MemPrO,^22^ e.g., -u POPE:7 -u POPG:2 -u CARD:1 -l POPE:7 -l POPG:2 -l CARD:1. The default is a bilayer of pure POPC. If custom composition is used, both leaflets must be specified.
-C/--conc: sets the NaCl concentration.
-mp/--mempro: passes additional arguments to MemPrO,^22^ such as grid points or minimization steps (default: 5 grid points, 15 minimizations).
-at/--cg2at: passes arguments to CG2AT-CCD2MD for atomistic conversion.

Environment variables required for MemPrO^22^ include NUM_CPU, PATH_TO_INSANE, and PATH_TO_MARTINI. For setup details, refer to the MemPrO GitHub.^22^

#### Outputs

Intermediate files include.

- OUTPUTNAME_nomem.pdb: nonmembrane-embedded system.
- CG files for atomistic systems.
- topol.top and relevant.itp files for GROMACS^19^ simulation.

For atomistic systems, water is represented using TIP3P. Topology files are generated by CG2AT-CCD2MD for AA systems and by Martinize2 and MemPrO^22^ for CG systems. Note that topology and.itp files may overwrite existing files in the working or output directories if the same output name is reused.

#### Special Case: at2mem

For embedding atomistic systems originally in CHARMM ordering, we use at2mem. The usage is similar to that above, but the -mem flag is only used to pass membrane composition and may be omitted.

#### Examples

Figure 3 shows membrane-embedded systems predicted using the userCCD option in AF3. Both AA and CG representations are shown for transmembrane proteins (first and second rows): Kir2.2,^30^ AAC,^31^ β_2_AR,^32^ and WaaL;^33^ and for a membrane-associated protein (bottom row): Grp1,^34^ PTEN,^35^ ClsA,^36^ and PSD.^37^

**Figure 3:**
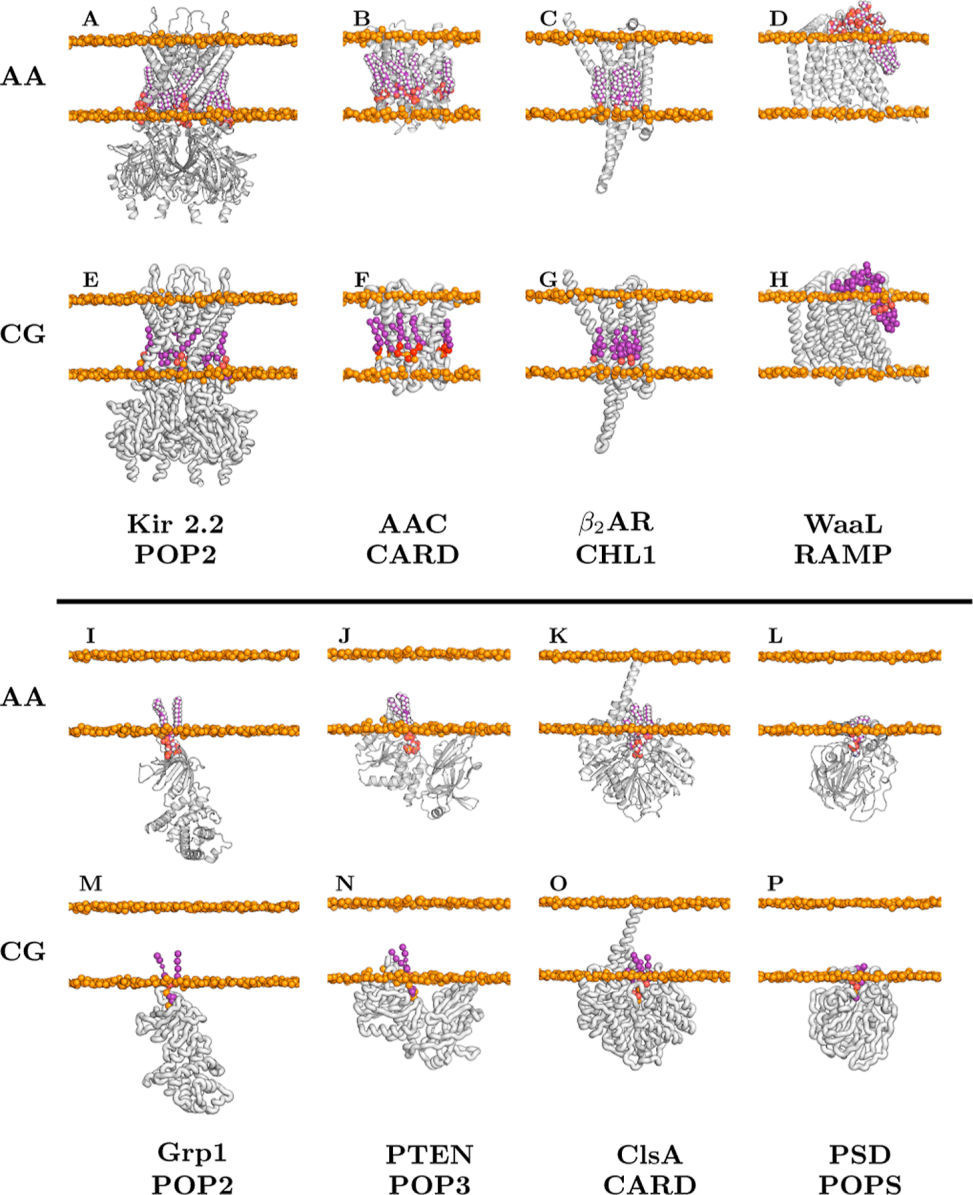
Protein–ligand complexes from AlphaFold3 ^10^ utilizing user-defined CCD codes (created via CCD2MD) for integral (above the line) and peripheral (below the line) membrane proteins. Each complex is presented in both atomistic (CHARMM, first and third rows) and coarse-grained (Martini, second and fourth rows) representations, supporting multiscale modeling of protein–ligand interactions. This figure illustrates a variety of membrane bound (first and second rows) and membrane associated (third and fourth rows) protein–lipid interactions of biological relevance. In (**A** (atomistic) and **E**) (coarse-grained), Kir2.2 (UniProt ID: Q14500) is shown bound to POPI-3,4-bisphosphate. (**B** and **F**) feature AAC (P05141) with cardiolipin, while (**C** and **G**) present β_2_AR (P07550) interacting with cholesterol. (**D** and **H**) depict WaaL (P27243) complexed with RAMP (LPS, see Table 1). In (**I** and **M**), Cytohesin-3 (O43739) is shown with POPI-3,4-bisphosphate, and (**J** and **N**) show PTEN (P60484) bound to POPI-3,4,5-trisphosphate. (**K** and **O**) present ClsA (P0A6H8) with cardiolipin, and (**L** and **P**) show PSD (P0A8K1) interacting with POPS.

### Dependencies

CCD2MD can be downloaded from GitHub: github.com/keb721/CCD2MD. All components of the package run in Python 3 and require only standard libraries along with two widely used, open-source packages: NumPy^38^ and Pandas,^39^ both of which are distributed under permissive licenses.

For atomistic conversion, GROMACS^19^ is required. For coarse-grained (CG) conversion and membrane embedding, Martinize2 ^28^ is also required. To use CIF files directly, version 0.14.0 of Martinize2 and the PyCifRW package[Fn fn1] are requirements. For membrane embedding, both MemPrO^22^ and CG2AT-CCD2MD^29^ are required; both are available in the CCD2MD GitHub repository.

## Discussion and Conclusions

The binding of ligands, particularly lipids, to proteins can significantly influence protein behavior.^1,2^ However, many membrane proteins contain lipid-binding sites whose functions remain poorly understood.^40^ Investigating these interactions is therefore a promising avenue for extending our understanding of membrane protein function.

Co-folding techniques represent a major advancement in the prediction of ligand-bound protein structures. CCD2MD builds on this by providing an extensible, open-source package that transforms cofolding outputs into simulation-ready systems. While the ligand database presented in this work focuses primarily on protein–lipid models and simulations using CHARMM and Martini force fields, the methodology is generalizable to other ligands and force fields. CCD2MD also supports user-defined SMILES strings, enabling compatibility across multiple cofolding platforms without requiring manual renaming of ligand outputs. We encourage users to extend the framework to incorporate ligands relevant to their own research. Although the majority of the ligands natively present in CCD2MD are lipids, we emphasize that the user-extensible framework in terms of the creation of user-CCD codes and additional mappings (see Supporting Information) allows this technique to be used for any generic ligand–including nucleic acids and metal ions, as well as a greater diversity of small molecules. We welcome user contributions of mappings of use to the community (mappings of CCD codes proceed similarly to those of SMILES strings; see Supporting Information).

Nevertheless, cofolding techniques are not without limitations. Chirality issues, especially with ligand representations, are common,^10,13,14^ even when using CCD codes.^15^ Although conversion to CG and back to atomistic can help mitigate these issues, it can also introduce slight shifts in atomic positions. Changing the cofolding program used may also improve results – we note the presence of ABCFold^41^ which allows for easy visual comparison of the outputs of AF3,^10^ Boltz-1,^12^ and Chai-1 ^11^ from the same multiple-sequence alignment. While CCD2MD can convert CCD outputs from all of these cofolding programs, there may be limitations if SMILES strings are used (see Supporting Information).

Future development plans include defining flags for typical membrane compositions, creating a pipeline for direct simulation of outputs, and distributing CCD2MD via PyPI for easier installation and integration. We also intend to extend the suite to allow for conversion to additional force fields of use to the biomolecular simulation community, although we highlight that the use of alternative atomistic force fields can be accommodated by the use of user-defined CCD codes via pos2cif.

## Supplementary Material



## Data Availability

The data generated by the simulations reported in this manuscript are openly available, along with the code for CCD2MD in the GitHub repository https://github.com/keb721/CCD2MD

## Footnotes

a
https://github.com/jamesrhester/pycifrw/.

## Notes

Details for creating an arbitrary SMILES mapping for use with CCD2MD (PDF)
