# Supplementary material for: CCD2MD: A Suite of Packages for Preparing Co-Folded Outputs for Molecular Dynamics Simulations
Source: J Chem Inf Model. 2025 Nov 5;65(22):12145–54. doi: 10.1021/acs.jcim.5c02066 (PMC12648654; doi:10.1021/acs.jcim.5c02066)
Supplement: Supplementary file 1 [file ci5c02066_si_001.pdf]

Supporting Information

CCD2MD: A Suite of Packages for Preparing Co-Folded Outputs for Molecular Dynamics Simulations

Katarina E. Blow,[*,†] Matyas Parrag,[†] and Phillip J. Stansfeld[*,†,‡]

†*School of Life Sciences, Gibbet Hill Campus, University of Warwick, Gibbet Hill Road, Coventry, CV4 7AL, UK*

‡*Department of Chemistry, University of Warwick, Gibbet Hill Road, Coventry, CV4 7AL, UK*

E-mail: katarina.blow@warwick.ac.uk; phillip.stansfeld@warwick.ac.uk

Additional mappings

In some cases, the use of AlphaFold 3[1] is not desirable, meaning that user-defined CCD codes cannot be created. Instead, ligands not present in the CCD database must be incorporated via SMILES strings. Many co-folding programs, including Chai-1[2] and Protenix,[3] name SMILES atoms sequentially by element type. This allows a single SMILES mapping to be reused across these platforms. In contrast, Boltz-1[4] assigns atom names using RDKit[1], requiring a unique mapping for each SMILES string.

Adding new molecules only requires knowledge of the mapping between ligand atoms. However, due to the non-uniqueness of SMILES strings, a single canonical SMILES string must be

[1]Boltz-1 GitHub, issue 110: https://github.com/jwohlwend/boltz/issues/110

defined for use with CCD2MD mappings. For example, the SMILES string used for POPE is:

```
CCCCCCCC\C=C/CCCCCCCC(=O)O[C@H](COC(=O)CCCCCCCCCCCCCCC)COP(O)(=O)OCCN
```

Minor modifications to this string (e.g., chirality changes or removal of double bonds) are permissible as long as the atom order remains unchanged. While it is technically possible to map multiple SMILES strings to the same molecule, this is not currently supported for CG conversion.

SMILES atom naming in reference files (e.g., `POES_CCD.txt`) follows conventions used by AF3[1] and Protenix:[3] the element symbol followed by a 1-indexed ID (e.g., `C1`, `C2`, `O1`, ...). This naming is compatible with Chai-1's[2] current (`C1_1`, `C2_1`, ...) and older (`C_1`, `C_2`, ...) conventions. When using SMILES strings, users must ensure that atom ordering in the co-folding input matches the mapping specified in CCD2MD reference files.

Each unique SMILES string must be assigned a SMILES name that links it to CHARMM and Martini mappings. SMILES names should not overlap with CCD codes or CHARMM residue names, as this causes processing issues. For CG conversion, SMILES names must not exceed four characters.

Creation of mappings for CCD codes proceeds in a similar manner, but does not require the addition of a `-S/--SMILES` flag in usage.

Usage in CCD2MD To use SMILES strings with `ccd2at` or `ccd2cg`, the SMILES names must be passed in the same order as they were input into the co-folding program using the `-S/--SMILES` flag. All SMILES ligands must be added sequentially. For example, if three POPG molecules (SMILES name `POGS`) and one NDP1 molecule (`NP1S`) were added in that order, the command would be:

```
-S POGS POGS POGS NP1S
```

Alternatively, the same input can be specified as:

```
-S POGS 3 NP1S
```

This assumes that all three POPG SMILES strings were added before the NDP1 string. SMILES names must be unique and consistent with the mappings defined in CCD2MD, and should not conflict with existing CCD or CHARMM names.

An example workflow using the POES SMILES string is presented in Figure S1.

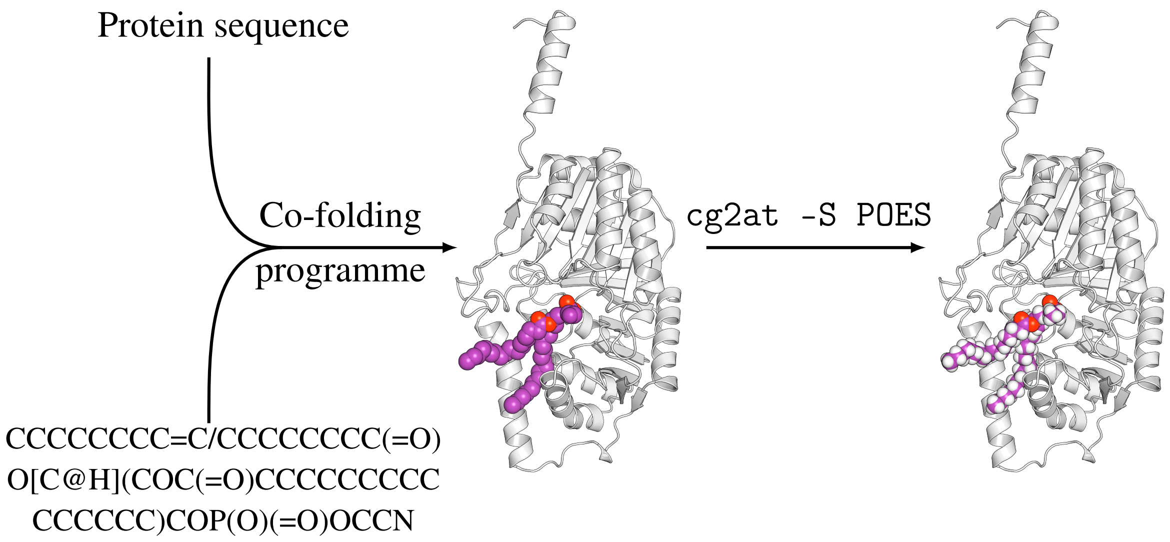

Figure S1: Example workflow for SMILES strings enabled by CCD2MD. Protein sequences (e.g., ClsB, UniProt ID: P0AA84) and ligand SMILES code, POPE are input into a co-folding program (e.g. AlphaFold[1]) to generate an atomistic structural prediction. Through the `-S` flag, the SMILES string can be processed through CCD2MD – here `ccd2at` is shown without membrane embedding but the pathway remains the same for coarse-graining or membrane embedding.

References

(1) Abramson, J. et al. Accurate structure prediction of biomolecular interactions with AlphaFold 3. *Nature* **2024**, *630*, 493–500.

(2) (Chai), C. D.; Boitreaud, J.; Dent, J.; McPartlon, M.; Meier, J.; Reis, V.; Rogozhonikov, A.; Wu, K. Chai-1: Decoding the molecular interactions of life. 2024.

(3) Team, B. A. A. et al. Protenix – Advancing structure prediction through a comprehensive AlphaFold3 reproduction. 2025.

(4) Wohlwend, J.; Corso, G.; Passaro, S.; Reveiz, M.; Leidal, K.; Swiderski, W.; Portnoi, T.; Chinn, I.; Silterra, J.; Jaakkola, T.; Barzilay, R. Boltz-1: Democratizing biomolecular interaction modeling. 2024.
